# Extramedullary Plasmacytoma Causing Myelopathy Resulting in Gait Imbalance

**DOI:** 10.7759/cureus.15171

**Published:** 2021-05-22

**Authors:** James Wert, Aimen Farooq, Anum Jalil

**Affiliations:** 1 Internal Medicine, AdventHealth Orlando, Orlando, USA

**Keywords:** radiotherapy (rt), cd 138 immunostain, extramedullary plasmacytoma, international myeloma working group, spinal cord compression

## Abstract

Plasmacytomas are neoplasms of plasma cells that can involve the bone marrow, the bone itself, or soft tissue without bone marrow involvement. Extramedullary plasmacytomas are most commonly found in the upper respiratory tract in a total of 80% of all cases. Extramedullary plasmacytomas have also been documented in locations such as the central nervous system. This form of plasmacytoma may be seen as a solitary entity or in patients with multiple myeloma. We present a case of a 66-year-old female with a history of multiple myeloma on maintenance therapy with lenalidomide who has been experiencing gait imbalance for the past two months. The patient had a thoracic MRI done that revealed a mass at T7-8 with associated cord compression. She was taken for surgical intervention by neurosurgery. Pathology revealed trabecular bone and cartilage infiltrated by sheets of plasma cells highlighted by immunostaining CD138, monoclonal lambda light chains, consistent with a plasma cell neoplasm (plasmacytoma). The patient’s hospital course was complicated by hypotension resulting in transient ischemic myelopathy that was addressed in the neuro-intensive care unit. The patient was then discharged to an inpatient rehabilitation center. She would follow up with her primary oncologist for localized radiation therapy.

## Introduction

Plasmacytomas may occur in the presence of other features of multiple myeloma or as a single lesion (solitary plasmacytoma), and more commonly involve bony rather than soft tissue (or extramedullary) sites. Of note, extramedullary multiple myeloma has a higher incidence in patients less than 55 years of age. The most commonly implicated anatomic locations of plasmacytomas include bony sites such as the vertebrae, ribs, sternum, and skull [1]. Extramedullary disease is estimated to occur in approximately 7% to 18% of patients with multiple myeloma at the time of diagnosis and in up to 20% of patients on follow-up, and is associated with a worse prognosis [2]. Soft tissue involvement from a hematogenous spread can occur in any organ, including skin, lymph nodes, liver, kidney, or the central nervous system. 

This case highlights the importance of interdisciplinary decision making as well as the need to be mindful of the patient’s past medical history. Extramedullary plasmacytoma is rare in this age group and, the diagnosis was arrived at by carefully considering the patient’s presenting symptoms, physical exam findings, and her known history of multiple myeloma. Collaboration and communication amongst specialties was the key in treating this patient with a complex medical history, and unusual presentation. 

## Case presentation

**Figure 1:**
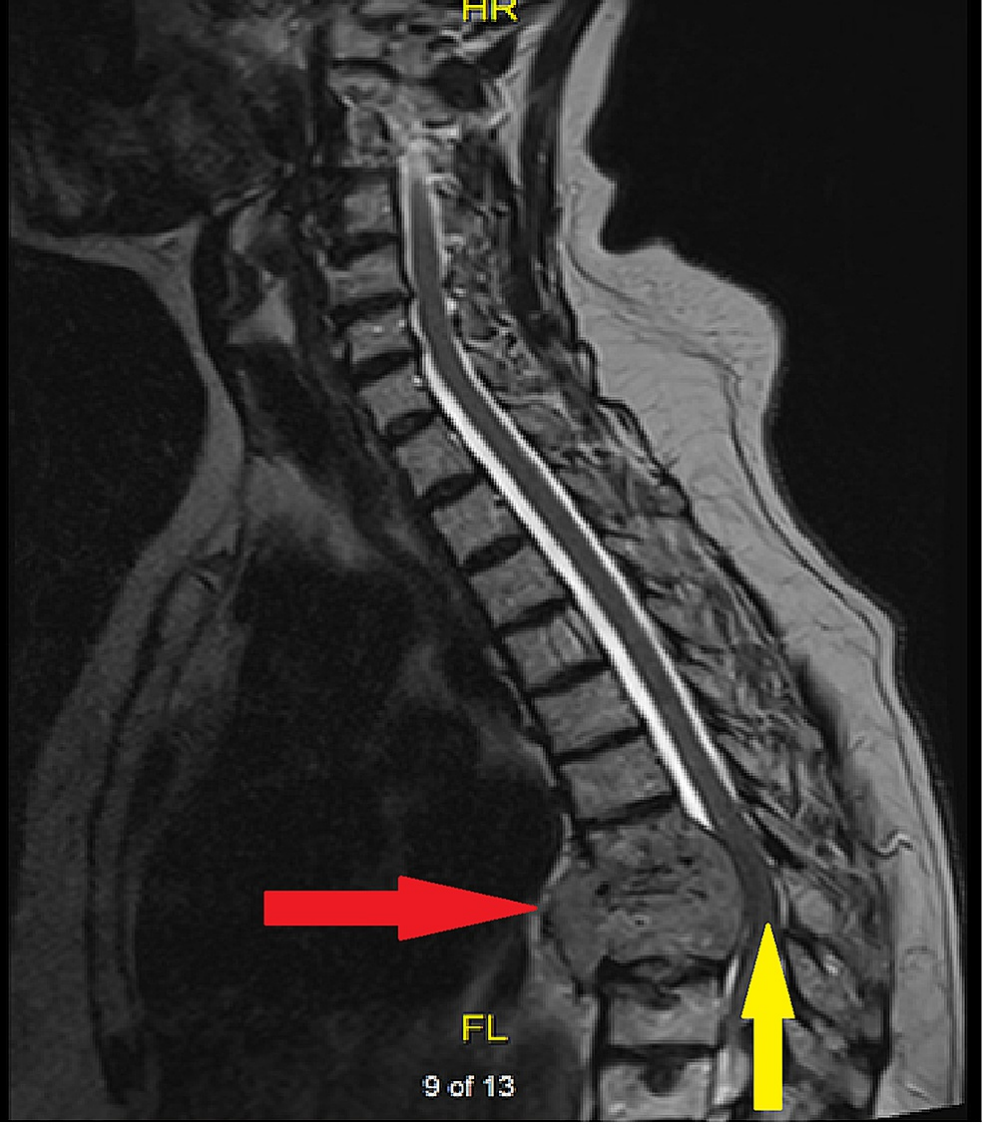
Plasmacytoma with spinal cord compression. Sagittal view of thoracic spine via T2 MRI. Red arrow indicating large plasmacytoma at the level of T7-T8. Yellow arrow indicating lack of contrast indicating cord compression as a result of the plasmacytoma

**Figure 2:**
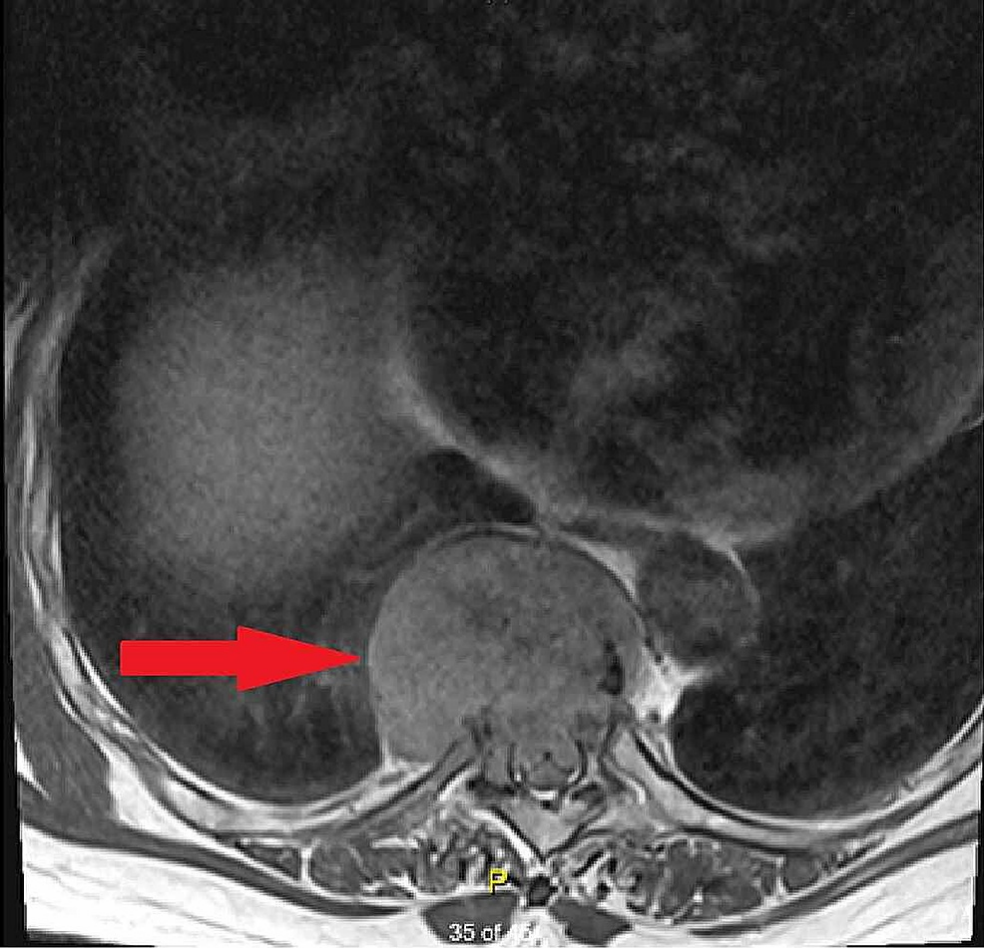
Plasmacytoma at the level of T8. Transverse view of thoracic spine via T2 MRI. Red arrow denoting the plasmacytoma within the central nervous system causing spinal cord compression

A 66-year-old female with a past medical history of essential hypertension, and multiple myeloma diagnosed in 2013, presented to the hospital with complaints of unsteadiness on her feet for the past two months. The patient noticed that she was having difficulty walking as a result of her legs feeling heavy. She also felt unsteady on her feet feeling like she was going to fall to her right side. She also experienced associated symptoms of numbness and tingling in her legs. Review of symptoms was otherwise negative except as mentioned. She has been on lenalidomide since 2013 for maintenance therapy for multiple myeloma. Her symptoms were progressive and persistent so she was initially seen by her primary care physician and was prescribed prednisone 80 mg orally for 10 days as well as a recommendation to be seen by her oncologist. Her oncologist ordered an MRI of thoracic spine which revealed a mass at T7-8 with associated cord compression as seen in Figures 1-2. Pertinent oncologic history was significant for a diagnosis of multiple myeloma with associated lumbar spine compression fracture that was surgically repaired in 2013. 

On initial evaluation in the emergency department (ED), the patient was found to be afebrile 97.5℉, hypertensive with a blood pressure of 194/78, tachycardia of 101, and saturating 100% on room air. She was in mild discomfort at the time of examination. She was tachycardic, lungs were clear to auscultation bilaterally, neurological exam was notable for a positive Romberg test, plantar and patellar reflexes were 3+ bilaterally, and she had a positive Babinski sign. Her initial lab work was unremarkable with a hemoglobin of 14.2 g/dl (normal range: 12.0-15.5 g/dl for women), a creatinine of 0.77 mg/dl (normal range: 0.6-1.2 mg/dl), and a vitamin B12 level of 204 pg/mL (normal range: 232-1245 pg/mL). A CT head without contrast was done in the ED, with no evidence of intracranial hemorrhage but it did reveal evidence of lytic lesions involving the calvarium consistent with the patient’s known history of multiple myeloma. Neuro-surgical consult was immediately obtained on admission due to concerning finding of cord compression. In the ED she received intravenous dexamethasone, intravenous antihypertensives, and was transferred to the neuro-surgical floor.

After neuro-surgical evaluation, she was scheduled for surgical intervention. The next day the patient was taken to the operating room where she underwent a T6-T10 laminectomy with spinal cord decompression, and bilateral transpedicular T7 and T8 corpectomies with resection of tumor.

Postoperatively, the patient was transported to the neuro-ICU. Her post-op physical exam revealed hip flexor strength of 4/5 in the morning. Later in the afternoon the patient became hypotensive with a blood pressure of 73/34 mmHg (MAP of 43 mmHg) and developed a change in her hip flexor strength of 2/5. There was concern for ischemic myelopathy secondary to hypotension. The patient was bolused with intravenous normal saline, blood cultures were drawn and intravenous antibiotics were empirically started. Phenylephrine was started with a goal MAP of greater than 80 mmHg. Neurosurgery was updated on the change in the patient's condition and recommended starting IV dexamethasone. After the patient’s blood pressure improved, her symptoms of lower extremity weakness also significantly improved; subsequently, she was weaned off phenylephrine.

**Figure 3:**
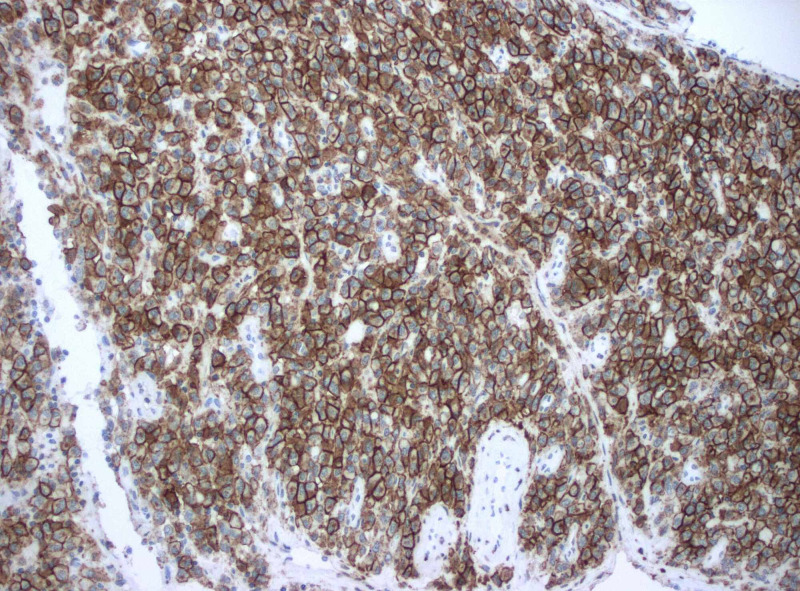
CD138 immunostaining showing trabecular bone and cartilage infiltrated by sheets of plasma cells

**Figure 4:**
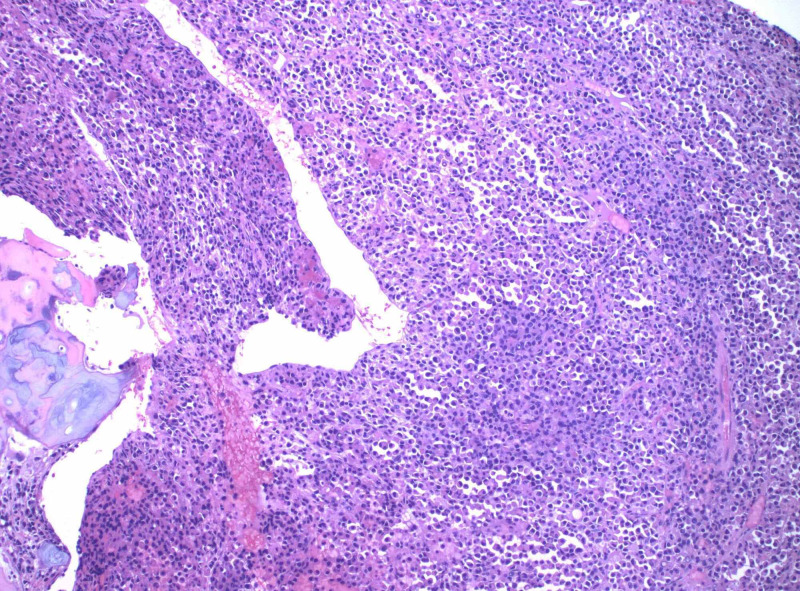
Hematoxylin and eosin stain of a plasmacytoma showing sheet like growth of malignant cells

**Figure 5:**
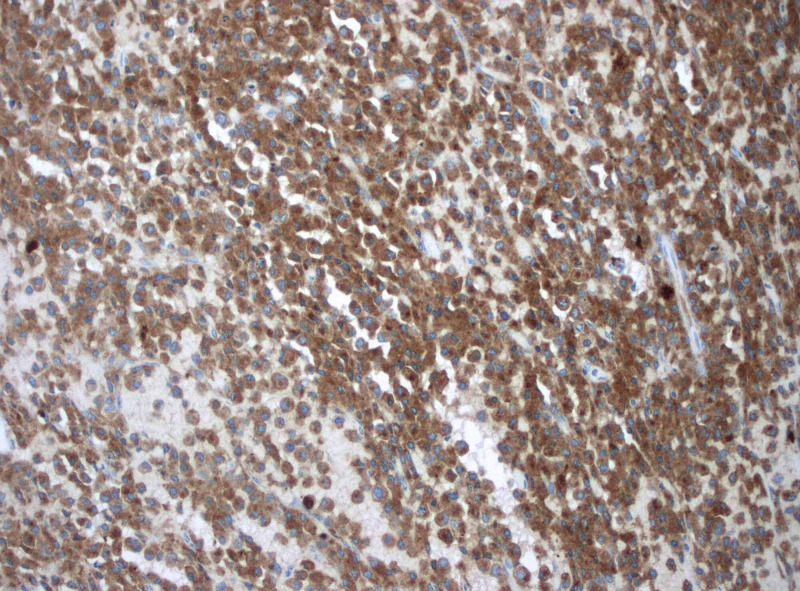
Lambda light chain immunostaining of a plasmacytoma revealing monoclonality

At this time during the patient’s hospitalization, the pathology report was available revealing trabecular bone and cartilage infiltrated by sheets of plasma cells highlighted by immunostaining CD138, monoclonal lambda light chains, consistent with a plasma cell neoplasm (plasmacytoma) as evidenced by Figures 3-5 [3]. She was evaluated by a radiation oncologist for localized radiation therapy that the patient declined as she wanted to receive the treatments at a facility closer to where she lived. She was discharged to an inpatient rehabilitation with plans to follow up with her oncologist and start localized radiation therapy to the area of resected plasmacytoma at T8.

## Discussion

Plasmacytomas are a clonal proliferation of B cells that were first described in 1905 by Schridde [4]. Extramedullary plasmacytomas comprise less than 4% of all plasma cell tumors [4]. The median age of presentation is 55-60 years with a male to female ratio of 3:1 [4]. Plasmacytomas can be differentiated into three categories that are solitary plasmacytoma of bone, extramedullary plasmacytoma, and multiple primary or recurrent plasmacytomas [5].

The diagnosis requires evidence of clonal plasma cells in a single site, without or minimal bone marrow involvement (<10% infiltration by plasma cells), absence of other lesions on skeletal survey, and end-organ damage as seen in symptomatic multiple myeloma (anemia, renal insufficiency, hypercalcemia, multiple bone lesions) [6]. The diagnosis of solitary or extramedullary plasmacytoma is made based on the diagnostic criteria established by the International Myeloma Working Group [7,8].

Extramedullary plasmacytomas are rare neoplasms that involve soft tissues without systemic involvement [9]. These neoplasms typically occur in the upper respiratory tract and oral cavity unlike the scenario described in this report which involved the central nervous system. Our case discusses a patient with multiple myeloma in remission with relapse as evidenced by pathology revealing plasmacytoma. Treatment should be deliberated on amongst a multidisciplinary team that includes a medical oncologist, radiation oncologist, pathologist, and primary care physician.

Biopsy and histological examination is needed to make a definitive diagnosis [10]. Aside from the pathology report, additional testing should be considered. Laboratory studies that should be ordered include serum and urine electrophoresis, quantitative immunoglobulin and beta-2-microglobulin in serum. Imaging studies may include computed tomography, MRI, and 18F-fluorodeoxyglucose-positron emission tomography [7].

In our case, the patient’s symptoms prompted advanced imaging that led to the finding of a mass at T7-T8. Neurosurgical consultation was obtained due to concerning symptoms related to cord compression as well as for tissue diagnosis. Advanced imaging including MRI of the thoracic spine was necessary to make the diagnosis and guide management options [11]. Pathology was indicative of an extramedullary plasmacytoma.

What will be seen on pathology slides are monoclonal plasma cells without B cell components. Monoclonality is important to establish as this makes the diagnosis. Immunophenotypic markers that can be tested for are CD138, CD38, kappa/lambda light chain ratio, CD19, CD56, CD27, CD117, and cyclin D1 [4]. Extramedullary plasmacytomas are diagnosed via biopsy [12].

Plasmacytomas are very sensitive to radiotherapy which remains the first-line treatment [13]. Response rates to radiotherapy for solitary plasmacytomas are excellent at 83% to 96% [6]. The recommended dose is fractionated radiotherapy at a total dose of 40-50 Gy over 4-5 weeks in daily doses [6]. The treatment field should extend an additional 2-cm to involve healthy tissue. Response rates in extramedullary plasmacytomas are even better at 80% to 100% with localized radiotherapy [6]. The entire tumor site, in addition to healthy surrounding tissue, is irradiated. Radiation doses vary but typically range from 35 to 60 Gy for a total of 20 fractions [6].

Surgery, either partially or completely, is usually a part of the initial workup. Extramedullary plasmacytomas are understood to have non-specific imaging findings with surgical excision typically required prior to definitive histologic diagnosis [14]. Surgically resected tissue are sent for pathology to make the diagnosis. Surgery is not the definitive treatment as radiotherapy remains the mainstay. Adjuvant chemotherapy has been studied with no beneficial effect [15,16].

## Conclusions

Extramedullary plasmacytomas are rare and even more so when located within the central nervous system. Although it is a rare diagnosis, it can have significant clinical manifestations for patients such as gait imbalance described in this case presentation. It is necessary to take a thorough history and physical exam to guide the next steps which in this case included appropriate advanced imaging with an MRI of the thoracic spine. Once the mass was located and given the patient's symptoms, she was taken to the operating room by the neurosurgical team. The mass was sent for pathology and revealed trabecular bone and cartilage infiltrated by sheets of plasma cells highlighted by immunostaining CD138, monoclonal lambda light chains, consistent with a plasma cell neoplasm (plasmacytoma). She required rehabilitation after her surgery to gain back strength and conditioning. Outpatient radiation therapy is coordinated with the radiation oncologist. With an uncommon presentation of a rare diagnosis such as extramedullary plasmacytoma, communication and interdisciplinary rounds are essential to providing high-quality care.
